# Successful third haploidentical hematopoietic stem cell transplantation after two graft failures in a pediatric patient with severe aplastic anemia: a case report with five-year follow-up

**DOI:** 10.3389/fimmu.2025.1607926

**Published:** 2025-07-09

**Authors:** Xuewei Li, Wenhui Zhang, Saisai Li, Xiaolin Ma, Xue Shi, Wei Wang, Lingjie Sun, Kuan Qiu, Yanxia Zhao, Chunting Zhao, Xiaodan Liu

**Affiliations:** ^1^ Department of Hematology, The Affiliated Hospital of Qingdao University, Qingdao University, Qingdao, Shandong, China; ^2^ Department of Bone Marrow Transplantation, The Affiliated Hospital of Qingdao University, Qingdao University, Qingdao, Shandong, China; ^3^ Department of Respiratory and Critical Care Medicine, The Affiliated Hospital of Qingdao University, Qingdao University, Qingdao, Shandong, China; ^4^ Children’s Medical Center, Department of Pediatric Hematology and Oncology, The Affiliated Hospital of Qingdao University, Qingdao University, Qingdao, Shandong, China

**Keywords:** severe aplastic anemia, hematopoietic stem cell transplantation, graft failure, immune response, conditioning regimens

## Abstract

**Background:**

Hematopoietic stem cell transplantation (HSCT) offers a potentially curative option for severe aplastic anemia (SAA). However, graft failure (GF) remains a life-threatening complication following HSCT. Haploidentical HSCT may serve as an effective salvage therapy for the treatment of GF.

**Case presentation:**

This report describes a 3-year-old girl with acquired SAA who experienced GF twice following matched unrelated donor (MUD) transplantations. Successful engraftment was ultimately achieved through a third haploidentical donor HSCT. This work was conducted in accordance with the Declaration of Helsinki and the Declaration of Istanbul.

**Conclusions:**

Based on our experience with this case, we conclude that a third HSCT with a haploidentical donor represents a viable approach to extending survival.

## Introduction

Severe aplastic anemia (SAA) is an autoimmune disorder characterized by the destruction of hematopoietic components in the bone marrow, leading to life-threatening complications such as infections and hemorrhage (1). The primary treatments for SAA include intensive immunosuppressive therapy (IST) and allogeneic hematopoietic stem cell transplantation (allo-HSCT). Compared to IST, allo-HSCT provides multiple advantages, including higher long-term survival rates (70%–80%), faster hematopoietic recovery, a reduced incidence of clonal diseases, and an improved quality of life. Therefore, HSCT from an HLA-matched related donor (MRD) is regarded as the first-line treatment for children with SAA (2). Graft failure (GF) following HSCT can manifest as either the failure of donor cells to achieve initial engraftment or the loss of donor cells after successful engraftment. Due to persistent leukopenia and thrombocytopenia, which significantly increase the risks of infection, bleeding, and related mortality (3), GF remains a life-threatening complication of HSCT. The cumulative incidence of GF is significantly higher in nonmalignant disorders compared to malignant ones, with an incidence ranging from 3% to 5% in patients with SAA (4). A very small proportion of patients with GF may achieve complete autologous recovery (5). For the majority, a second transplantation remains the only potentially curative option (6). Despite favorable recovery of neutrophil counts, a second transplantation is associated with a high rate of non-relapse mortality (NRM) (7). However, a third allo-HSCT has rarely been reported. This report describes a pediatric patient with SAA who experienced GF twice following matched unrelated donor (MUD) transplantations. The patient successfully achieved engraftment following a third haploidentical HSCT and remained with clinical remission during a subsequent 5-year follow-up, highlighting the effectiveness and safety of this approach.

## Case presentation

A 3-year-old girl was admitted to our hospital with pancytopenia. Complete blood count revealed: leukocyte count 1.89 × 10^9^/L, neutrophil count 0.01 × 10^9^/L, hemoglobin 68 g/L, reticulocyte count 0.0015 × 10¹²/L, and platelet count 1 × 10^9^/L. Multisite bone marrow (BM) examinations demonstrated markedly hypocellularity. The results of the sternal puncture show a significant reduction in BM hyperplasia, with severely reduced myeloid hyperplasia and severely reduced erythroid hyperplasia, and slightly varying sizes in the erythroid precursors. Lymphoid hyperplasia is significantly active, accounting for 90%. The marrow comprises an empty reticular structure, mostly consisting of non-hematopoietic cells. No megakaryocytes were observed in the entire section, and platelets are relatively rare. The karyotype was 46, XX. Assessment of peripheral blood flow cytometry for CD55 and CD59 was negative, which excludes paroxysmal nocturnal hemoglobinuria (PNH). The chromosome breakage test was negative. The coombs and lactate dehydrogenase were normal. Genetic testing for inherited bone marrow failure syndromes (IBMFS) was performed through a comprehensive panel analysis encompassing Fanconi anemia (FA) and related disorders. No pathogenic germline variants were detected through this investigation.

She was prescribed combination therapy with cyclosporine A (CsA) and danazol. At the six-month follow-up, the patient was diagnosed with SAA accompanied by persistent hypocytosis. She had a 9/10 human leukocyte antigen (HLA)-matched unrelated donor identified, with matches at the HLA-A, -B, -DRB1, and -DQB1 loci, except for a mismatch at the HLA-C locus. The donor, a 49-year-old male, was also ABO-compatible with our patient. Cytomegalovirus (CMV) and epstein-barr virus (EBV) immunoglobulin G (IgG) were positive, while immunoglobulin M (IgM) was negative. The patient underwent the first allo-HSCT six months after being diagnosed with SAA. HLA class I and class II antibodies were negative. The conditioning regimen consisted of fludarabine (FLU, 30 mg/m² for 4 days), cyclophosphamide (CTX, 30 mg/kg/day for 4 days), and rabbit anti-thymocyte globulin (r-ATG, 10 mg/kg, Thymoglobulin^®^, Sanofi Corporation, France), collectively referred to as the FCA conditioning regimen. The patient was transfused with peripheral blood stem cells (PBSC) containing mononucleated cells (MNC, 6.62×10^8^/kg) and CD34^+^ cells (4.47×10^6^/kg). The excess cells (MNC, 25.53×10^8^/kg, CD34^+^ cells, 20.56×10^6^/kg) were cryopreserved at −80°C. During the first HSCT, a total of 8 units of red blood cells and 9 therapeutic doses of platelets were transfused. Graft-versus-host disease (GvHD) prophylaxis was administered using CsA, short-term methotrexate (MTX, 15 mg/m² on day 1 and 10 mg/m² on days 3, 6, and 11), and mycophenolate mofetil (MMF, 0.25 g orally twice daily from day −7 to day 30 post-HSCT). Neutrophil engraftment was achieved on day 12 post-HSCT (+12), followed by platelet engraftment on day +13. Nonetheless, the patient developed pancytopenia with progressive mixed chimerism of 87.68% on day +23. Due to the low CsA concentration (89.8 ng/mL), the CsA dosage was increased to maintain a blood concentration of 150–250 ng/mL. On day +83, pancytopenia has not improved, accompanied by stable mixed chimerism at 86.81%. Bone marrow (BM) examination revealed severe hypocellularity and signs of cytomegalovirus (CMV) infection associated with GF. CMV-DNA levels increased to 5 × 10³ copies/mL, prompting the addition of ganciclovir, granulocyte colony-stimulating factor (G-CSF), and thrombopoietin (TPO) to the treatment regimen. However, on day +123, the pancytopenia showed no improvement despite CMV-DNA being undetectable, with mixed chimerism declining to 30.68%.

After donor-specific antibodies (DSA) were confirmed to be negative, the patient underwent a second allo-HSCT on day 154 following the first HSCT. The conditioning regimen for the second MUD-HSCT was adjusted to include fludarabine (FLU, 30 mg/m² for 4 days), busulfan (Bu, 0.8 mg/kg every 6 hours for 2 days), CTX (50 mg/kg for 2 days), and r-ATG (7.5 mg/kg, from the same manufacturer). GvHD was prevented using CsA (maintained at 150–250 ng/mL), MMF, and short-term MTX as previously described. The patient received the unfrozen PBSC from previously matched unrelated donor (HLA 9/10) containing CD34^+^ cells and MNC at doses of 9.32 × 10^6^/kg and 9.67 × 10^8^/kg, respectively. Before the second transplantation, rituximab (375 mg/m²) was administered on day −11 to address a slight increase in Epstein-Barr virus (EBV) levels (2×10³ copies/mL). The patient received 8 units of red cell transfusions and 20 units of platelets. Neutrophil recovery was achieved on day +13, and platelet engraftment occurred on day +15, with no signs of GvHD.

Unfortunately, on day 21 after the second HSCT, the patient developed pancytopenia again, accompanied by a progressive decline in mixed chimerism to 94%. Pancytopenia progressed, accompanied by stable mixed chimerism at 59.11% on day 71 after the second HSCT. Despite administering adequate doses of immunosuppressive agents, the patient experienced GF again. The dose of infused PBSC from the initial donor was increased with CsA prophylaxis of GvHD, which containing peripheral blood mononuclear cells (PBMC, 1.02×108/kg) containing CD34+ cells (1.17×106/kg) and CD3+ cells (0.8×107/kg) without the induction of GvHD. Chimerism initially increased to 91.28% but rapidly declined to 13.44% by day 79 after the second HSCT, with no improvement in pancytopenia. BM aspiration after GF exhibited a reduction in BM hyperplasia, with reduced myeloid hyperplasia and erythroid hyperplasia. Lymphoid hyperplasia was active, accounting for 63%. No megakaryocytes were observed in the entire section, and platelets are relatively rare.

**Figure 1:**
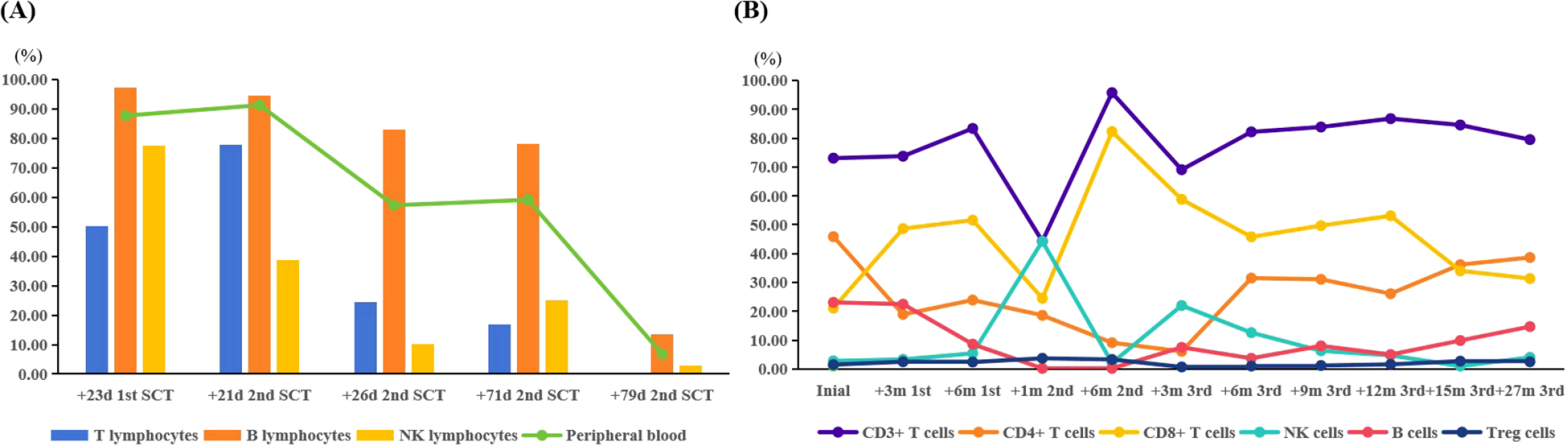
**(A)** The chimerism of the different lymphocyte subsets during the decrease in donor chimerism. **(B)** The immune reconstitution following the three transplants. SCT, stem cell transplantation; +23d 1st SCT, on day 23 post the first SCT; +21d 2nd SCT, on day 21 post the second SCT; +26d 2nd SCT, on day 26 post the second SCT; +71d 2nd SCT, on day 71 post the second SCT; +79d 2nd SCT, on day 79 post the second SCT; +3m 1st, 3 months post the first SCT; +6m 1st, 6 months post the first SCT; +1m 2nd, one month post the second SCT; +6m 2nd, 6 months post the second SCT; +3m 3rd, 3 months post the third SCT; +6m 3rd, 6 months post the third SCT; +9m 3rd, 9 months post the third SCT; +12m 3rd, 12 months post the third SCT; +15m 3rd, 15 months post the third SCT; +27m 3rd, 27 months post the third SCT.

B cells, T cells, and natural killer (NK) cells were isolated from peripheral blood (PB) using magnetic bead-based sorting, and donor-derived chimerism of each cellular subset was independently assessed. As depicted in **Figure 1A**, following the first two allo-HSCT, the patient demonstrated robust donor-derived B-cell chimerism while exhibiting relatively lower levels of T-cell and NK-cell chimerism. Donor-derived chimerism in PB exhibited a significant decline, with concomitant marked reductions in donor chimerism across all three magnetically sorted cellular subsets (B cells, T cells, and NK cells). Following the third allo-HSCT, donor-derived chimerism in PB, B cells, T cells, and NK cells reached stable levels, with all cellular subsets consistently exceeding 95% for durable engraftment. Notably, the patient demonstrated suboptimal donor-derived immune reconstitution following the first two allo-HSCT, characterized by delayed lymphopoiesis and insufficient functional maturation of T/NK cell subsets. In contrast, following the third allo-HSCT, robust immune reconstitution was observed, with donor-derived B cells, T cells, and NK cells achieving substantial repopulation by post-transplant month 6 and subsequently maintaining stable levels consistent with durable engraftment criteria (**Figure 1B**).

**Figure 2:**
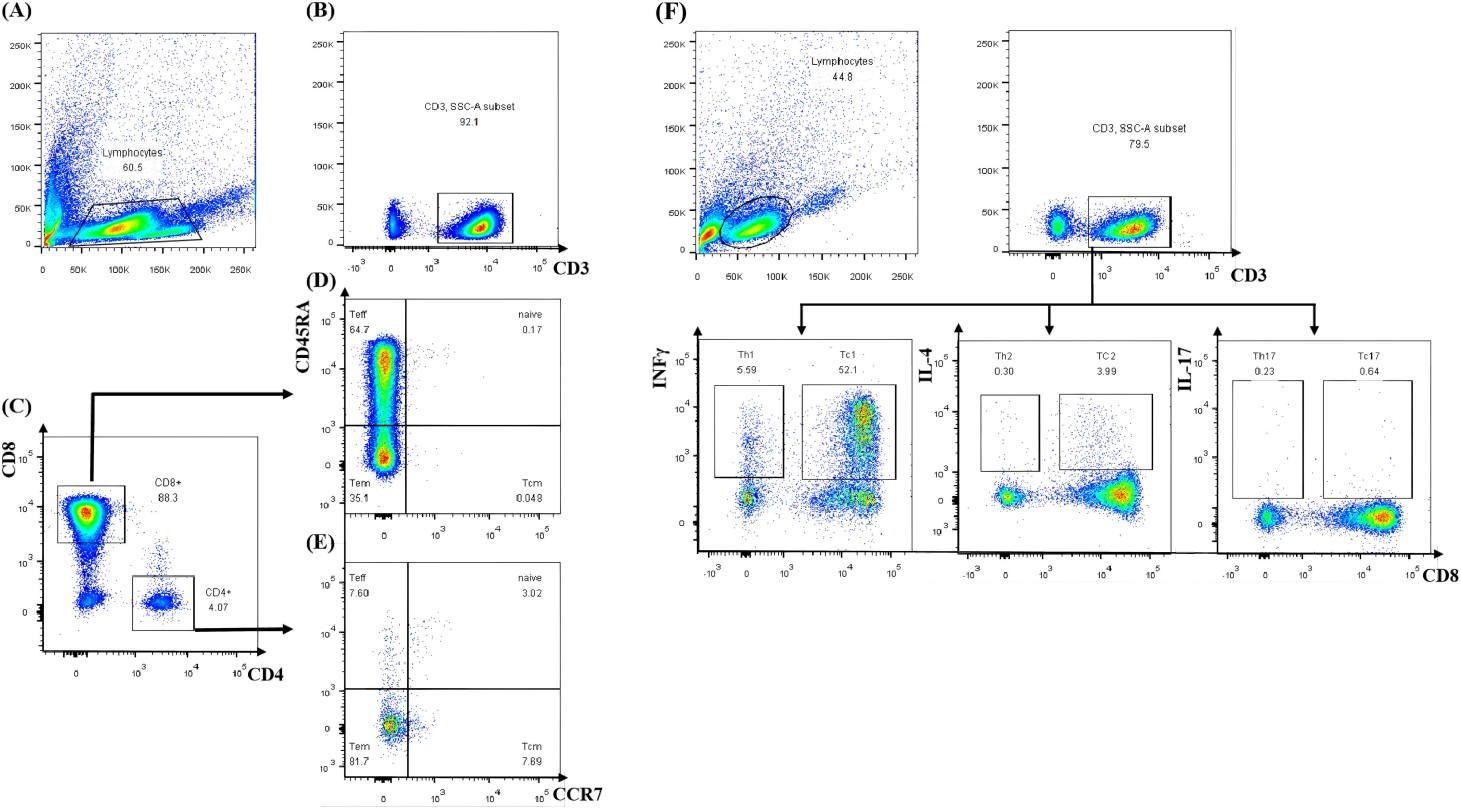
Lymphocyte subset analysis by flow cytometry. **(A)** Total lymphocytes. **(B)** CD3+ T cell selection. **(C)** CD4+/CD8+ T cell subsetting. **(D, E)** Memory/effector phenotype profiling. **(F)** T cell subset cytokine secretion analysis by flow cytometry. Teff, terminally differentiated effector T cell; Tem, effector memory T cell; Tcm, central memory T cell.

**Figure 3:**
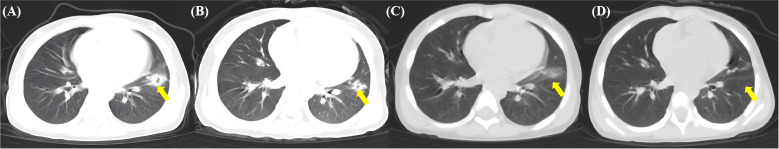
The yellow arrow indicates the location of the invasive pulmonary fungal infection. **(A)** Left lung nodule with ground-glass opacity (GGO) and halo sign on day 22 after the third haplo-SCT. **(B)** The nodule lesion and GGO have diminished on day +40. **(C)** The nodule lesion and GGO have resolved, evolving into fibrotic streaks on day +60. **(D)** The fibrotic streaks have further attenuated and diminished, with lesions substantially resolved on day +90.

A third HSCT was considered due to persistent pancytopenia, recurrent infections, and transfusion dependency. Lymphocyte subset analysis of both the donor and recipient was performed. It was indicated that interferon-gamma (IFN-γ) was significantly elevated in the patient after the second GF (**Figure 2**). After DSAs were confirmed to be negative, the patient underwent the third allo-HSCT 194 days after the second transplant. The conditioning regimen for the third haplo-HSCT included total body irradiation (TBI, 3 cGy for one day), fludarabine (FLU, 30 mg/m² for 4 days), CTX (35 mg/kg for 4 days), and r-ATG (7.5 mg/kg, from the same manufacturer). In the transplantation protocol of this study, a sequential infusion strategy was adopted: donor BM stem cells were infused on day 1, and PBSC were infused on day 2. The cumulative infused cell dose was: MNC 9.67×10^8^/kg, CD34+ cells 9.32×10^6^/kg. Additionally, we incorporated the post-transplant cyclophosphamide (PTCy, CTX 14.5mg/kg on days +3 and +4, respectively) into the third haplo-HSCT to prevent GvHD. Neutrophil and platelet engraftment were achieved on day +13 and day +15, respectively. An invasive pulmonary fungal infection occurred on day +22 and was successfully treated with voriconazole, achieving resolution on day +90 (**Figure 3**). Intravenous immunoglobulin (IVIG, 5 g, equivalent to 0.4 g/kg) was administered twice a week for a duration of 6 months, and no infections were detected. Routine screening includes assessments of growth, endocrine function, pulmonary function, bone health, and cancer screening to ensure early detection and timely treatment of potential late effects.

Currently, the patient has remained disease-free for 5 years, with a high quality of life, normal growth and development, and no signs of GvHD, infections, thyroid dysfunction, or cataracts, demonstrating the convincing effectiveness and safety of this approach. This work complied with the Declaration of Helsinki and the Declaration of Istanbul.

## Discussion

The latest expert consensus recommends stratified management of SAA treatment based on the spectrum of patient age, best donor availability, disease status, and treatment response (8). HSCT from a matched related donor (MRD), associated with high overall survival (OS) and event-free survival (EFS), is considered a first-line therapy for children and young AA patients, attributed to qualitative advantages over IST (2, 8). However, only a small number of SAA patients have MUD. Experts currently propose that allo-HSCT from a well-MUD could also be considered (8). Iftikhar et al. also endorsed increased utilization of HCT by prioritizing matched unrelated or haploidentical donor HCT over IST in children and adults who lack a matched related donor (9), but the randomized clinical trial of IST versus MUD for pediatric patients is still ongoing (10). The ideal unrelated donor is a male or a nulliparous female, HLA-matched donor at the A, B, C, DRB1 loci, younger than 30 years of age, and CMV-compatible with the recipient. In our case, the donor was a 49-year-old male with a 9/10 HLA match, ABO blood group compatibility, and CMV compatibility with the recipient. HSCT was performed using an FCA conditioning regimen, with an adequate dose of PBSC. Unfortunately, GF occurred after each HSCT from this donor.

Primary GF (pGF) remains a rare but life-threatening complication following allogeneic hematopoietic stem cell transplantation and is a significant contributor to morbidity and mortality (11–13). It was characterized by the absence of initial donor cell engraftment (donor cells <95%), absolute neutropenia (ANC ≤0.5×10^9^/L), anemia (hemoglobin <80g/L), and thrombocytopenia (platelets <20×10^9^/L) by day+28 post-allo-HSCT using either mobilized PBSC or BM progenitors in the absence of relapse (7). For umbilical cord blood transplantation, these criteria apply by day +42 due to the expected delayed engraftment of umbilical stem cells (7, 14). In contrast, secondary GF refers to the progressive loss of donor cell function following successful engraftment of donor hematopoietic stem cells and hematopoietic reconstitution in the recipient. This condition is characterized by a subsequent decline in blood cell counts, with persistent absolute ANC ≤ 0.5 × 10^9^/L and PLT< 20 × 10^9^/L (7). The incidence of pGF ranges from 0.8% to 20% (15), varying significantly depending on the method of T-cell depletion. GF results from the recipient’s immune response against donor immunohematopoietic cells, driven by residual host immunity that remains active despite the conditioning regimen. Residual host T cells are considered the primary effector cells responsible for rejection (16). It have been reported that an increased risk of GF in cases of HLA mismatch (17) and major ABO mismatch (18). Notably, the identification of donor-specific anti-HLA antibodies (DSAs) has been recognized as a major cause of pGF in haploidentical HSCT (19–23). However, donor cytotoxic T cells play a facilitative role in HSC engraftment. The absence of donor T cells in blood and bone marrow due to graft T-cell depletion is associated with an increased incidence of GF (3). Moreover, CD4^+^CD25^+^Foxp3^+^regulatory T cells (Tregs) and Natural killer (NK) cells also play a role in graft failure pathogenesis (24). In patients with engraftment dysfunction and pGF, elevated serum levels of IFN-γ, decreased Th2 and Tc2 cells, and imbalances in the Th1/Th2 and Th17/Treg ratios may contribute to the pathogenesis of engraftment dysfunction (2, 3, 25, 26). GF may be related to insufficient immune reconstitution, inadequate tolerance due to the patient’s compromised immune function, or abnormalities in the BM microenvironment. These factors collectively impair the recipient’s immune system’s ability to effectively support the engraftment and growth of the transplanted HSCs. Studies have shown that children with GF exhibit significantly elevated serum levels of IFN-γ, and CXCL9 compared to control groups. Furthermore, the use of an anti-IFN-γ monoclonal antibody, such as emapalumab, has been demonstrated to facilitate successful engraftment in second transplants using the same donor (27). Alternatively, it may result from other mechanisms, such as drug toxicity, sepsis, or viral infections, including CMV, human herpesvirus 6 (HHV-6), and parvovirus (3). In our case, the patient experienced two times of GF with the same unrelated 49-year-male-donor, despite receiving adequate HSCs and a different conditioning regimen for the second HSCT. She had not received chemotherapy prior to HSCT, received an adequate dose of HSCs, and did not develop graft-versus-host disease. We analyzed lymphocyte subsets in both the donor and recipient and found no abnormalities in lymphocyte subpopulations, including CD4^+^ T cells, CD8^+^ T cells, and Tregs. However, elevated levels of IFN-γ were detected in the patient, suggesting the involvement of immune-related factors.

The OS of patients with GF can be enhanced through interventions such as cytokine therapy, IST, DLI, ATG, alemtuzumab, and a second HSCT. A study on serial chimerism analysis in patients with acquired AA highlights an important distinction from the standard approach used following HSCT for leukemia. In leukemia, immunosuppression is typically withdrawn in response to declining donor chimerism. However, current guidelines for acquired AA recommend continued serial chimerism monitoring and the reinstatement of IST when donor chimerism decreases post-HSCT (28). The management of patients experiencing GF following HSCT has historically included reinfusion of cryopreserved progenitor cells (29) or administration of high-dose hematopoietic growth factors (30). However, both approaches have been associated with poor outcomes in cases of true GF. DLI from an alternative donor has been shown to effectively address secondary graft failure by eliminating residual host cells through immune-mediated mechanisms and improving chimerism (31). In our case, intensified IST and treatment with hematopoietic growth factors proved ineffective. Despite DLI temporarily increasing chimerism to 91.28%, it rapidly declined to 13.44%, indicating the need for salvage treatment. Following the failure of an HLA-matched graft, the original donor is typically preferred for a second transplantation, as identifying a similarly matched secondary donor is often challenging (32–35). A second HSCT provides the best opportunity for long-term survival in patients with primary or secondary GF. In this case, the patient underwent a secondary HSCT using unfrozen PBSC from the original donor, but GF recurred shortly thereafter.

A third HSCT was deemed the only viable option for the patient due to transfusion dependency and recurrent infections. In the absence of an MRD, her father was chosen as the donor for the third HSCT. A multicenter study on refractory severe AA revealed that haplo-HSCT achieved a 94% engraftment success rate, with a 3-year OS rate of 89% and a failure-free survival (FFS) rate of 86.8%, compared to unrelated donor HSCT (36). They also reported on 52 children who underwent haplo-HSCT (37), with 29 patients receiving HSCT as a salvage treatment and the remaining 23 patients as a front-line therapy. No significant differences were observed between the two groups across most clinical endpoints, including myeloid engraftment time (*P*=0.175), grade II–IV acute GvHD (*p*=0.699), chronic GvHD (*p*=0.916), OS (*p*=0.698), and FFS (*p*=0.899). Furthermore, it has been recently reported that 268 of 275 evaluable patients (97.5%) obtained sustained full donor chimerism, and 93.4% had complete hematopoietic recovery during the long-term follow-up (38). Improved allo-HSCT outcomes with unrelated and haploidentical donors are reflected in the panel’s recommendation of allo-HSCT using MRD or MUD as the preferred initial therapy in younger, medically fit patients (8). The panel also endorses increased utilization of HSCT by prioritizing matched unrelated or haploidentical donor HSCT over immunosuppressive therapy in children and adults who lack a matched related donor (9). Thus, haplo-HSCT can be considered a viable alternative treatment option.

In addition, BM contains not only CD34+ cells but also supportive components such as BM stromal cells and mesenchymal stem cells (MSCs), which differ from PBSC in surface markers and differentiation potential. Bone marrow-derived stem cells may more readily home to the bone marrow microenvironment, while peripheral blood stem cells show stronger proliferative activity (39, 40). Their combination covers a broader hematopoietic stem cell subset spectrum and enhances engraftment success. Patients with AA often exhibit BM microenvironmental abnormalities. Meanwhile, BM-derived MSCs exhibit immunosuppressive functions, capable of inhibiting recipient T-cell activation and proliferation, reducing transplant rejection, and promoting stem cell homing. Moreover, it has been established that BM should be the preferred stem cell source for matched sibling transplants in acquired AA in patients of all age groups (41, 42). In our case, although a substantial number of PBSC and CD34+ stem cells were infused during the first two HSCTs, both resulted in GF, likely due to the abnormal BM microenvironment. Therefore, the patient underwent haplo-HSCT and received BM and PBSC from her father in the third HSCT. Engraftment was achieved rapidly, and no GvHD was observed.

Despite various conditioning regimens and T-cell depletion strategies had been evaluated, the optimal approach for a third transplantation in AA remains undefined. The optimal conditioning regimen appears to be a combination of fludarabine and CTX or melphalan with radiation therapy, as it is associated with higher engraftment rates and improved survival outcomes (43). When combined with partial T-cell depletion, TBI-containing regimens have demonstrated improved OS compared to regimens using CTX alone (44). Nonetheless, the significance of low-dose TBI in HLA-mismatched related SCT remains unknown (45, 46). Younger children who undergo TBI are considered to be more vulnerable to late effects such as gonadal and thyroid dysfunction, growth impairment, and secondary malignancies (47). It has been reported that 11 of 13 (85%) patients transplanted before age 3 with TBI had neuropsychological abnormalities (48). Given the very young age of our patient and concerns about the late effects, we did not include TBI in the first two transplants. However, both methods have been proved inadequate for our patient, prompting the addition of TBI during the third HSCT. High-dose TBI-containing regimens have been shown to mitigate the increased risk of GF in unrelated-donor HSCT for pediatric acquired AA. Nonetheless, high-dose TBI regimens are associated with high rates of early toxicity as well as late effects compared to patients who do not receive TBI (49). A dose-evaluation study found that a TBI dose of 2–4 cGy, combined with CTX and ATG, was sufficient to facilitate engraftment without causing prohibitive toxicity in patients with an HLA-matched donor (50). This combination was associated with the highest survival rates following matched unrelated donor HSCT (MUD-HSCT). Kojima et al. analyzed data from 154 patients with SAA who underwent MUD-HSCT and identified ovarian dysfunction as a significant late complication (51). Based on these reports, we selected for low-dose TBI at 3 cGy and implemented ovarian shielding during TBI. The successful engraftment achieved with acceptable regimen-related toxicities indicated that this conditioning regimen was a feasible option for salvage haplo-HSCT in patients with SAA.

In clinical practice, it is recommended to switch to a different type of ATG for subsequent courses, such as horse ATG (h-ATG) if r-ATG was used initially, and vice versa. This strategy aims to minimize adverse events associated with ATG, particularly serum sickness, which tends to occur earlier and more severely upon re-treatment with the same brand of ATG (52). However, since 2007, h-ATG has become unavailable in most Asian, Latin American, and European countries, with r-ATG being the only available formulation (53). A repeat course of immunosuppression with the same formulation of r-ATG is rarely reported. In a study on secondary IST, serum sickness was observed in 2 of 39 patients during a median follow-up period of 283 days (54). In this case, we used r-ATG with the same formulation and manufacturer due to the unavailability of h-ATG. No serum sickness was observed, even after three courses. To minimize the risk of serious infections, we reduced the ATG dosage to 7.5 mg/kg during the second and third HSCTs, given the short interval between each transplantation. We monitored immune reconstitution following HSCT and observed a slow but steady recovery without any serious infections, suggesting that the dosage was appropriate.

Various protocols have been implemented to achieve sustained engraftment in unmanipulated T-cell-replete transplantation. Mixtures of granulocyte colony-stimulating factor (G-CSF) primed BM (G-BM) plus G-CSF mobilized peripheral blood grafts (G-PB) have successfully introduced T-cell hyporesponsiveness, promote polarization of T cells from Th1 to Th2, and provided protection against acute graft-versus-host disease (aGvHD) (55). Additionally, G-BM may include BM-derived mesenchymal stem cells (MSCs) in the graft, which could reduce the severity of GvHD and enhance engraftment (56). PTCy in the allo-HSCT setting has facilitated engraftment and resulted in GvHD rates comparable to those observed with MUD-HSCT in hematologic malignancies. Reducing GvHD is especially important in non-malignant conditions, where the goal is to achieve stable engraftment without excessive immune complications (57). Clay et al. reported a pilot study in which allo-HSCT using post-transplant successfully rescued 4 patients with AA who experienced primary GF after unrelated donor transplantation or umbilical cord blood transplantation (UCBT) (58). We use PTCy for GvHD prophylaxis, and no acute or chronic GvHD was observed.

## Conclusion

When a haploidentical donor is the only available option for a patient who experiences two episodes of GF after an alternative donor transplantation, a combination of BM and PBSC, conditioning regimens including fludarabine and low-dose TBI, and PTCy regimen for GvHD prophylaxis can improve the outcomes after the third HSCT. However, further investigations are necessary to establish more effective and safer allo-HSCT protocols for GF in patients with AA.

## Data Availability

The original contributions presented in the study are included in the article/supplementary material. Further inquiries can be directed to the corresponding authors.
